# Nonoxidative dehydrogenation of propane using boron-incorporated silica-supported Pt Sites synthesized by atomic layer deposition

**DOI:** 10.55730/1300-0527.3648

**Published:** 2023-12-04

**Authors:** Gökhan ÇELİK

**Affiliations:** Department of Chemical Engineering, Faculty of Engineering, Middle East Technical University, Ankara, Turkiye

**Keywords:** Propane, dehydrogenation, boron, platinum, atomic layer deposition, propylene

## Abstract

Nonoxidative dehydrogenation of propane to propylene using Pt-based supported catalysts is an active research area in catalysis because catalyst attributes of Pt sites can be controlled by careful design of active sites. One way to achieve this is by the addition of a second metal that may impart a change in the electron density of active sites, which in turn affects catalytic performance. In this study, bimetallic Pt and B sites were deposited on powder SiO_2_ using atomic layer deposition (ALD). Boron was first deposited on SiO_2_ via half-cycle ALD using triisoproplyborate as the B source. Following calcination, Pt deposition was performed via half-cycle ALD using trimethyl(methylcyclopentadienyl)platinum(IV) as the Pt source. The synthesized catalysts were reduced under H_2_ at 550 °C and characterized using inductively coupled plasma optical emission spectroscopy for elemental analysis, diffuse reflectance infrared Fourier transform spectroscopy of adsorbed CO to examine the properties of Pt, and time-resolved X-ray absorption near edge structure spectroscopy to examine the changes in the reducibility of Pt sites. The samples were then tested for nonoxidative dehydrogenation of propane at 550 °C using a fixed-bed plug-flow reactor to examine the role of B on the catalytic performance. Characterization results showed that the addition of B imparted an increase in electron density and affected the reducibility of Pt sites. In addition, incorporating B on SiO_2_ created anchoring sites for Pt ALD. The amount of Pt deposited on B/SiO_2_ was 2.2 times that on SiO_2_. Catalytic activity results revealed the addition of B did not change the initial activity of Pt sites significantly, but improved propylene selectivity from 80% to 87% and stability almost threefold. The enhanced selectivity and stability of PtB/SiO_2_ is most presumably due to favored desorption of propylene and mitigating coke formation under reaction conditions, respectively.

## 1. Introduction

With the advent of abundant shale gas resources and increasing demand for olefins worldwide, dehydrogenation of light alkanes emerged as a viable alternative to naphtha cracking to produce olefins. Olefins are building blocks for the chemical industry for producing many commodities, polymers, and chemicals. Among olefins, propylene is the second most produced light olefin and is used to produce polypropylene, propylene oxide, acrylonitrile, etc. Demand for propylene is increasing continuously, and on-demand production of propylene gains significance since conventional routes for producing olefins are energy-intensive, have low selectivity, and are unlikely to meet the anticipated demand [1–3].

Alkanes can be dehydrogenated to alkenes via different routes including oxidative dehydrogenation using strong oxidants such as O_2_ or mild oxidants such as N_2_O, H_2_O, or H_2_S [4–7], and nonoxidative dehydrogenation [1–3]. Oxidative dehydrogenation is a highly exothermic reaction, taking place at 425–475 °C. However, selectivity control in oxidative dehydrogenation due to uncontrolled oxidation reactions at high rates (production of CO_x_) is difficult, and that decreases the olefin yield. Nonoxidative dehydrogenation, on the other hand, is an endothermic and thermodynamically limited reaction requiring high operating temperatures (550–600 °C) to obtain industrially relevant catalytic rates. At these temperatures, catalysts suffer from sintering of active sites and coke deposition under reaction conditions [1–3]. Nevertheless, these issues could be overcome by catalyst design and engineering.

Nonoxidative dehydrogenation is performed over different catalysts including Pt-based catalysts, CrO_x_-based catalysts, and bulk metal oxide catalysts [8,9]. Among these, Pt-based catalysts have been studied extensively and preferred choice of catalysts because of their ability to perform the dehydrogenation selectively by facile C-H activation over C-C cleavage, despite the high cost and low abundance of Pt [3,10]. In addition, Pt-based catalysts are currently used in commercially employed dehydrogenation systems for on-purpose production of olefins: UOP Oleflex using PtSn/Al_2_O_3_, Dow using Pt/Ga/Al_2_O_3_, Flotu Tsinghua using PtSn/SAPO-34, and Sabic using PtSnK/SAPO-34 [2,11].

Accessible Pt sites catalyze not only the desired dehydrogenation reaction, but also the undesired concomitant side reactions such as cracking, isomerization, hydrogenolysis, and deep dehydrogenation. It has been reported that the former reaction is structure insensitive and can be catalyzed by all accessible Pt sites, whereas the large Pt ensembles catalyze the undesired side reactions, lowering selectivity and deactivating catalysts due to coke formation [12–15]. It also has been reported that the electronic and geometric properties of Pt-based catalysts play a significant role in the catalytic performance of the reactions involved in light alkane dehydrogenation, particularly, the side reactions [12,13]. These findings opened an avenue for catalysis researchers to obtain active, selective, and stable catalysts and to suppress the undesired side reactions by modifying catalyst attributes of Pt-based catalysts. A plausible way to achieve this is by synthesizing bimetallic catalysts via careful catalyst design. Many catalyst formulations have been tested for light alkane dehydrogenation, including, but not limited to, PtCu [16–18], PtCo [19,20], PtFe [19,21], PtGa [22], PtIn [23,24], PtMn [25,26], PtNi [19,27], PtZn [14,28–30], and found to improve catalytic performances.

Boron was previously used as an active support as well as a secondary element for bimetallic catalysts for the dehydrogenation of light alkanes [31–34]. For example, hexagonal boron nitride materials that had been known to be inert toward dehydrogenation showed catalytic activity for propane dehydrogenation with a high propylene selectivity and conversion [35]. In addition, in our earlier study, we demonstrated that B sites supported on SiO_2_ via liquid grafting were found to improve the stability of Pt-based n-butane dehydrogenation catalyst [33]. The catalyst was synthesized via sequential liquid grafting using organometallic B precursor B(OiPr)_3_ and Pt precursor ((MeCp)PtMe_3_) at room temperature with an intermediate calcination step under dry air at 450 °C. Investigations over used catalysts using characterization techniques such as X-Ray photoelectron spectroscopy and nuclear magnetic resonance showed that B sites deposited by liquid organometallic grafting facilitate the diffusion of carbon from Pt sites to B. PtB catalyst after n-butane dehydrogenation reaction was found to have less deposit of carbonaceous species than monometallic Pt catalysts. The main role of the B sites was to scavenge the carbon that deactivates the catalyst and extend the lifetime of the catalyst [33].

In this contribution, Pt and B containing bimetallic catalysts were prepared via sequential ALD. ALD is a self-limiting thin film growth technique that uses alternating exposures of pairs of precursor vapors on a solid surface [36,37]. At early cycles, the growth takes place via the formation of nanoparticles that possess well-defined catalytic sites and interfaces [38,39]. After ALD, the catalysts were characterized via in situ diffuse reflectance Fourier transform spectroscopy (DRIFTS) of adsorbed CO to examine the changes in the metal properties of Pt upon boron introduction, and time-resolved X-ray absorption near edge structure (XANES) spectroscopy–temperature programmed reduction (TPR) to examine the changes in the reducibility of Pt sites. The samples were then tested for nonoxidative dehydrogenation of propane at 550 °C using a fixed-bed plug-flow reactor to examine the role of B on the catalytic performance of propane dehydrogenation.

## 2. Materials and methods

### 2.1. Materials

Boron and platinum were deposited on commercial silica in powder form using a homemade viscous flow ALD reactor [38,40]. Prior to the deposition, silica was dehydroxylated at 200 °C for 2 h under a 0.06 torr vacuum. After the pretreatment, the temperature of the deposition furnace was reduced to 150 °C. Owing to the design of the reactor, this temperature was measured outside the deposition furnace with a thermocouple. The actual temperature at which the deposition takes place was measured in a separate experiment and found to be higher than the furnace temperature by 19 °C. Triisoproplyborate (B(OiPr)_3_, Sigma) in a stainless steel bubbler at room temperature was used as a B source [41]. One half cycle and 30 doses of B deposition were performed. After the B deposition was over, a sample was taken out from the furnace and calcined in a tubular furnace under dry air flow at 450 °C to burn the ligands and obtain boron oxide.

After the calcination, B-incorporated SiO_2_ was loaded into the ALD chamber and pretreated at 200 °C for 2 h. Then, the temperature was reduced to 90 °C (furnace temperature, the actual deposition temperature is around 117 °C, as measured in a separate experiment) for Pt deposition. Although the deposition of Pt using (MeCp)PtMe_3_ occurs at appreciably large growth rates between 200–300 °C, the deposition was performed with 30 doses of Pt precursor at 90 °C to slow down the growth and deposit the Pt sites with high dispersion [42,43]. Trimethyl(methylcyclopentadienyl)platinum(IV) ((MeCp)PtMe_3_, Strem Chemicals) in a stainless steel bubbler at 70 °C was used as a Pt source. Half cycle and 30 doses of Pt deposition were performed on B-incorporated SiO_2_. After the deposition, the sample was taken out from the deposition chamber and used in characterization experiments after reduction under H_2_ at 550 °C. The sample was denoted as PtB/SiO_2_. Pt/SiO_2_ was also synthesized without B-deposition, for comparison purposes.

### 2.2. Characterization

DRIFTS of Adsorbed CO: The nature of Pt nanoparticles was examined using CO as a probe molecule through in situ DRIFTS. Catalysts in fine powder form were loaded into the sample chamber of the in situ reaction cell (Harricks, Praying Mantis) which can achieve a controlled gaseous environment and temperature. The reaction cell with its optical accessory was placed in the sample compartment of an FTIR (Thermo Scientific Nicolet iS50 FTIR) instrument equipped with a liquid-N_2_ cooled MCT detector. The cell was first flushed with Ar at room temperature to sweep ambient air from the system. Then, the temperature was increased to 500 °C for reduction under H_2_ for 30 min. The sample was then cooled to 40 °C under Ar flow and a background spectrum was collected. Then, CO adsorption was started for 1 h. After the adsorption was over, the flow was switched back to Ar and sample spectra were acquired with respect to time and subtracted from the background spectrum collected prior to the CO adsorption at the same temperature.

Time-Resolved X-ray Absorption Near Edge Structure (XANES) Spectroscopy–Temperature Programmed Reduction (TPR): Pt LIII edge (11564 eV) XANES measurements in fluorescence mode were performed on the insertion device beamline of the Materials Research Collaborative Access Team (MRCAT-10ID) at Advanced Photon Source at Argonne National Laboratory. A cryogenically cooled double-crystal Si(111) monochromator was used with a Pt-coated mirror to minimize the presence of harmonics. XANES measurements were performed in quick-scan mode, with each scan taking approximately three min. XANES of a Pt foil was collected simultaneously with each sample measurement for energy calibration and alignment. The Pt edge energy was determined as the position of the maximum of the first peak in the first derivative of the XANES region.

Pt/SiO_2_ or PtB/SiO_2_ was placed in an X-ray absorption fine structure (XAFS) reaction cell that can achieve controlled gaseous environment and temperature. First, the spectrum reproducibility was checked by obtaining at least three scans under air before the time-resolved reduction under H_2_. Then, H_2_ flow was started, the temperature of the XAFS cell was increased at a rate of 5 °C/min, and data acquisition was started. The temperature of the cell was registered in the data files as well. Due to a communication problem in the reduction of PtB/SiO_2_ experiment, five scans were missed. The average oxidation state of Pt was obtained by fitting the XANES spectra as a linear combination of Pt foil and (MeCp)PtMe_3_. ATHENA software was used for the analysis. [44].

Inductively Coupled Plasma Optical Emission Spectroscopy (ICP-OES): Pt and B content of the synthesized catalysts were obtained by ICP-OES using a Perkin-Elmer Optima 4300DV ICP-OES.

### 2.3. Catalytic activity measurements

Catalytic activity measurements were performed using a ¼ in OD quartz fixed-bed plug-flow reactor. Catalysts in powder form were placed inside the middle of the reactor and supported from both ends using quartz wool. The reaction temperature is controlled via a K-type thermocouple located inside the reactor just above the quartz wool above the catalyst bed. The reactor assembly was then placed in a tubular furnace and connected to the catalytic activity system. The system was flushed with Ar to sweep the trapped air in the system and heated to the reaction temperature. Prior to the reaction, catalysts were reduced in situ at 550 °C under 30 sccm H_2_ for one h. The feed gas was then switched to 10 sccm of 7.1% Propane/Argon and the reaction started. The separation, detection, and quantification of reaction products as well as unconverted feed gas were performed with an online gas chromatography equipped with valves, a sulfated-alumina column, and a flame ionization detector (FID). Carbon balance was calculated to ensure the mass balance of the system and found to be accurate up to 97% or higher.

## 3. Results and discussion

Elemental Analysis: The table shows Pt and B content of the synthesized catalysts. The results revealed that the amount of Pt on PtB/SiO_2_ catalyst is significantly higher than that of Pt on Pt/SiO_2_. The higher loading of Pt on PtB/SiO_2_ catalyst is attributed to the creation of new anchoring sites associated with B deposition on SiO_2_ surface. Byron et al. also observed an increase on the amount of Pt sites grafted on SiO_2_ upon B-grafting. [33]. The authors performed sequential grafting of the same metals in hexane using the same precursors and found that Pt amount on B-grafted catalyst was 1.4 times that on B-free catalyst, as compared to 2.2 times difference by the results of this study. The authors attributed the increase in the Pt amount grafted on the surface to the improved anchoring on the B-sites. The authors supported their argument by density functional theory calculations, considering the adsorption energy of different fragments of Pt sites (i.e. Pt, Pt_2_, and MeCpPt) on a variety of boron-silica cage cluster models. They found that Pt sites have more affinity to interact with B-containing surface sites, with tetrahedrally coordinated boron sites being the most favorable for these interactions [33].

The amount of B deposited on PtB/SiO_2_ catalyst was found to be 0.68 wt%. This amount is slightly higher than the reported loading of 0.59% when the sequential grafting was performed in the liquid phase using the same organometallic precursor. Byron et al. stated that the amount of boron grafted on the surface did not change as the concentration of B in the grafting solution was increased and attributed this to surface saturation. Slightly higher loading of B of this study is not surprising since the deposition methods and grafting phase and temperature are different (liquid grafting vs vapor grafting), all of which could affect how much boron could be deposited on the surface. In addition, it is possible that multilayer formation of B sites may occur in ALD, causing an increase in the amount of B deposited.

**Figure 1:**
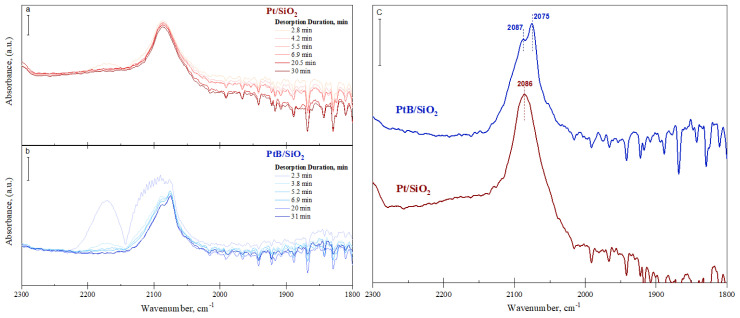
DRIFTS spectra of CO adsorbed on Pt/SiO_2_ and PtB/SiO_2_. (a) and (b) the change of absorbance with respect to desorption duration of Pt/SiO_2_ and PtB/SiO_2_, respectively, (c) the comparison of the spectra collected at circa 30 min.

DRIFTS of adsorbed CO: The nature of Pt nanoparticles on Pt/SiO_2_ and PtB/SiO_2_ was examined using CO as a probe molecule by infrared observation of vibrational properties of carbonyl species through in situ DRIFTS. These carbonyl species that formed on Pt sites describe the properties of Pt on B-free catalyst and the effect of boron on the nature of Pt [45–51]. To form carbonyl species, catalysts were first saturated with CO and purged with Ar in situ to remove weakly bound carbonyl species. Shown in Figures 1a and 1b are how the infrared absorbance spectra evolve with respect to the desorption of CO. The first spectrum collected just after the adsorption was over shows gas-phase CO IR band on PtB/SiO_2_. This band is anticipated because it takes time to completely flush CO in the cell with Ar. The gas CO IR band disappears with time, yet, the absorbance of other IR bands remains unchanged, indicating that carbonyl species remain on the surface after desorption and stable at 40 °C.

The Pt/SiO_2_ catalyst exhibited one band at 2086 cm^–1^ (Figure 1c) which was ascribed to linearly adsorbed CO on Pt sites [45]. In boron-containing catalyst, in addition to the IR band at 2087 cm^–1^, a new additional IR band was observed at 2075 cm^–1^, suggesting the formation of Pt sites with distinct properties than the ones in Pt/SiO_2_. This IR band observed on PtB/SiO_2_ with a red shift of 12 cm–^1^ originated from increased back-donation of Pt’s d-orbital electrons to the antibonding orbital of CO and attributed to an increased electron density of Pt sites [14,52,53]. Computational studies on PtB/SiO_2_ catalytic systems point out that the enhanced anchoring effect owing to the presence of B on SiO_2_ occurs on tetrahedral boron sites that are stabilized by Pt [33,54]. These BO_4_ sites may act as Bronsted acid sites and cause an increase in the stability of adsorbed species and electron density of Pt [33,54–56]. The increase of electron density is experimentally evidenced in our study by DRIFTS of adsorbed CO. However, the determination of the exact origin of the redshift requires further studies because other electronic and geometric effects may cause a similar shift in energy.

It should also be noted that the catalysts did not have any IR bands below 2000 cm^–1^ due to bridged CO adsorption, suggesting that Pt sites are highly dispersed without any major agglomeration [50,51].

**Figure 2:**
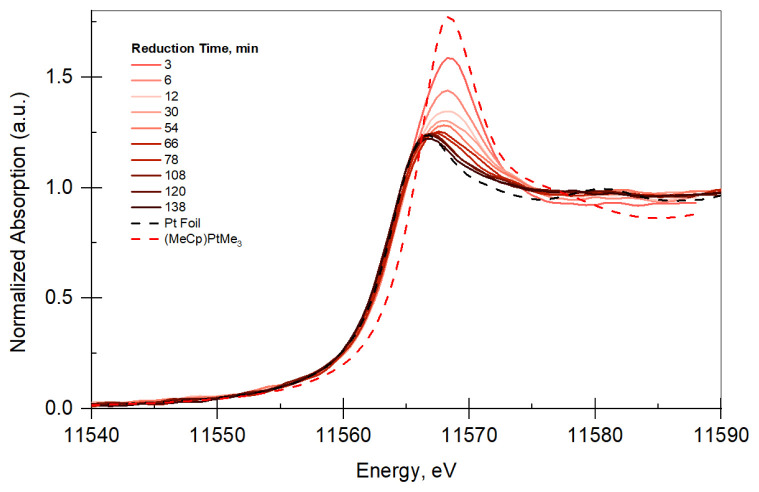
XANES plot for Pt/SiO_2_ (not all data shown for clarity). The legend shows the reduction time and the references used for XANES analysis.

**Figure 3:**
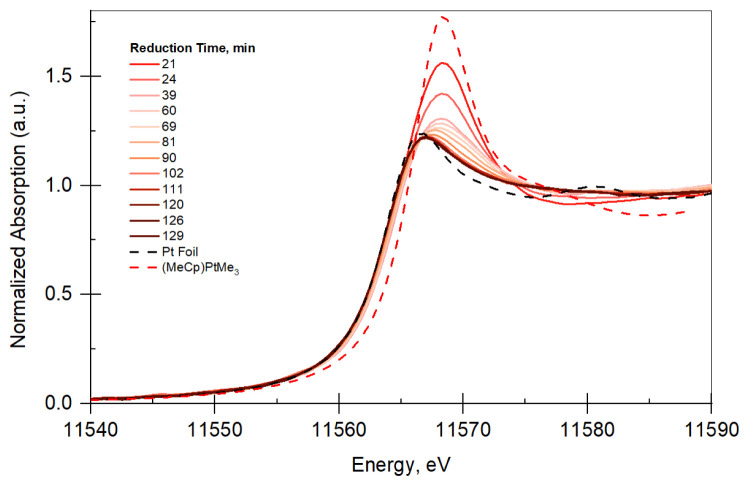
XANES plot for PtB/SiO_2_ (not all data shown for clarity). The legend shows the reduction time and the references used for XANES analysis.

Time-Resolved XANES TPR: Pt LIII edge (11.564 keV) XAFS measurements in fluorescence mode were performed. Owing to low metal loading, the samples did not give XAFS data with high signal to noise in transmission mode. Therefore, data were acquired in fluorescence mode. Measurements were performed under hydrogen while increasing temperature to obtain a reduction profile of Pt/B/SiO_2_ and Pt/SiO_2_. The temperature is increased at a ramp rate of 5 °C/min. The normalized absorption vs. Energy plots (XANES plots) for Pt/SiO_2_ and Pt/B/SiO_2_ are given in Figure 2 and Figure 3, respectively. Prior to the reduction, XANES of Pt/SiO_2_ and PtB/SiO_2_ were very similar to that of (MeCp)PtMe_3_, indicating that adsorbed species on the surface resemble the organometallic precursor used for the deposition. Both Figures show that XANES profile of the catalyst change from XANES of the precursor to that of Pt foil with respect to reduction time, indicating that Pt sites are reduced.

**Figure 4:**
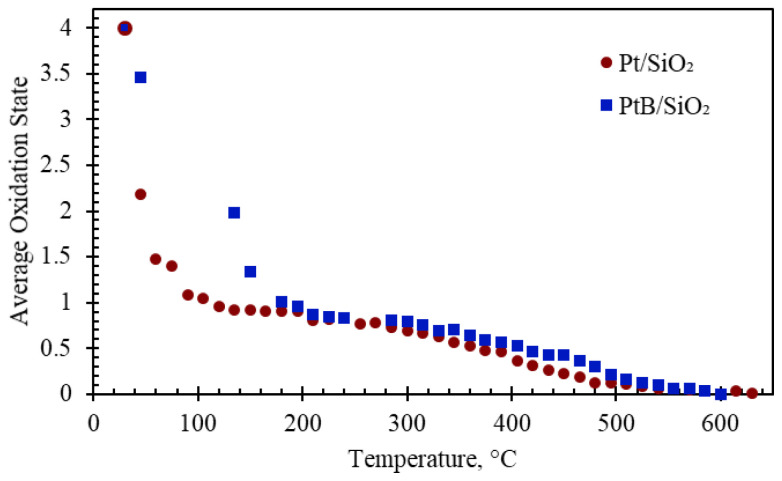
Average oxidation state with respect to temperature for Pt/SiO_2_ and PtB/SiO_2._

Figure 4 shows how the average oxidation state of Pt changes with respect to temperature. The desorption duration was converted to the reduction temperature, which was acquired during the experiment together with the spectra. XANES spectra were fitted using (MeCp)PtMe_3_ and metallic Pt foil. The first markers in Figure 4 indicate the average oxidation state of the samples before the reduction. The average oxidation state of Pt in both samples is +4 and the same as that of Pt in (MeCp)PtMe_3_. The XANES scans show that both samples reduce all the way to metallic Pt by 500 °C. The reduction of Pt sites on Pt/SiO_2_ underwent a large decrease (from 4 to 1) in the average oxidation state below 100 °C. The same extent of reduction for PtB/SiO_2_ did not occur until the reduction temperature of 180 °C. The rest of the reduction for both samples, however, exhibited a similar reduction profile. The decrease in the average oxidation state of Pt sites at low temperatures was observed on Pt-based catalysts synthesized via liquid grafting using the same organometallic precursor. Bunquin et al. attributed this to hydrogenolysis of the Pt-Me groups, originating from the deposited precursor [14]. The authors also observed an evolution of methane signal while the Pt-Me groups were hydrogenolyzed. [14]

The delayed reduction of Pt sites on PtB/SiO_2_ was attributed to the presence of B which retards the decomposition of intact precursor molecules after ALD. In other words, when B_2_O_3_ is present on the surface, it takes higher temperatures to hydrogenolyze the Pt sites. This is presumably due to the enhanced electron density of Pt sites, as evidenced by the red shift observed in DRIFTS of adsorbed CO, which could make interactions of (MeCp)PtMe_3_ with the B-containing SiO_2_ surface stronger. It is also possible that the presence of B on the surface may create new anchoring sites for ALD of (MeCp)PtMe_3_ and these sites may require larger energy for Pt-Me decomposition. The increase in Pt uptake (Table) is evidence that new anchoring sites are created on the B/SiO_2_. Based on the DRIFTS of adsorbed CO, it can be claimed that the strength of the platinum-support interaction of new sites is stronger.

**Figure 5:**
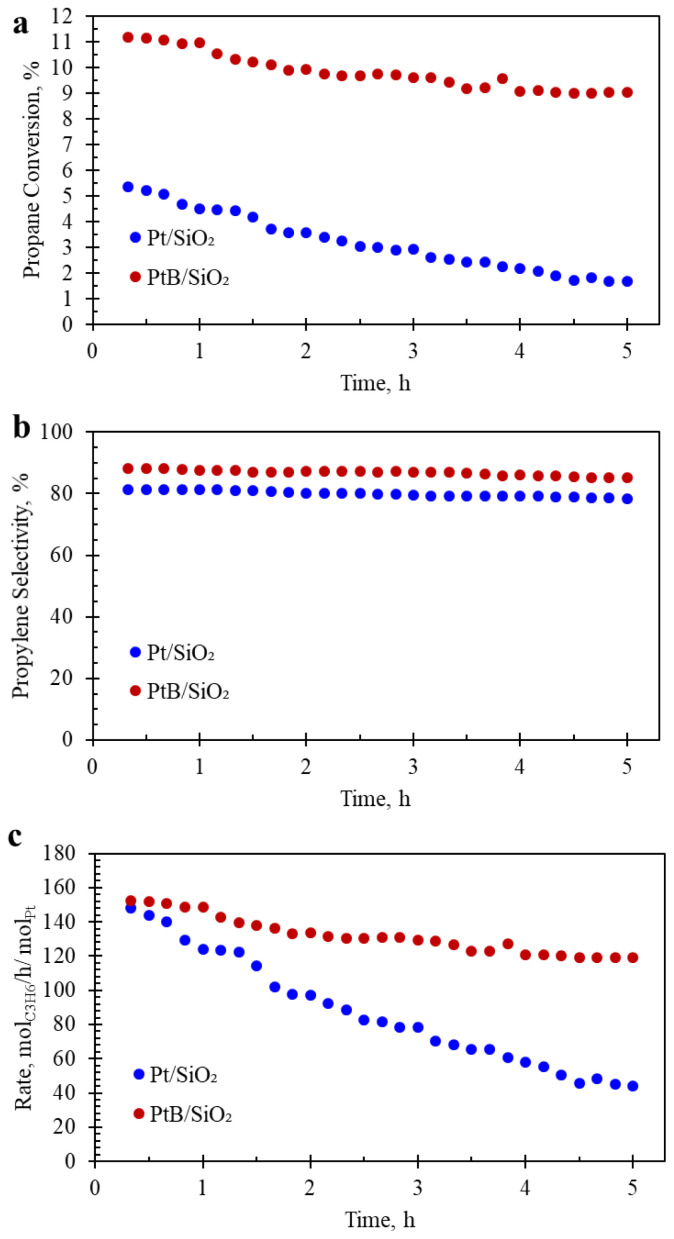
Nonoxidative dehydrogenation of propane catalytic activity results showing (a) propane conversion, (b) propylene selectivity, and (c) rate of propylene formation at an equal amount of Pt. Temperature: 550 °C, Flow Rate: 10 ccm of 7% Propane/Ar flow, Amount of catalyst = 200 mg. Bare silica and boron-incorporated silica did not show any appreciable catalytic conversion (< 0.18%) under the same conditions.

Catalytic Activity Results: PtB/SiO_2_ and Pt/SiO_2_ were tested in a fixed-bed plug-flow reactor for nonoxidative dehydrogenation of propane at 550 °C. The reactor was operated at low conversions and thus, can be considered as a differential reactor. Figure 5 shows propane conversion and propylene selectivity with respect to reaction duration. Initial propane conversions obtained over PtB/SiO_2_ and Pt/SiO_2_ (Figure 5a) were 11.2% and 5.4%, respectively. Examining the change of conversion with respect to time shows that PtB/SiO_2_ catalyst was more stable than Pt/SiO_2_. During the course of the reaction, propane conversion over PtB/SiO_2_ decreased from 11.2% to 9.1%, whereas propane conversion over Pt/SiO_2_ decreased from 5.4% to 1.7%, indicating that catalysts are under the influence of major deactivation causes. The deactivation constant, K_d_ (h^–1^), calculated via Eq.1 (x is the fractional conversion calculated at the start and the end of the reaction, and t (h–^1^) is the reaction duration) [3,14] for Pt/SiO_2_ (0.24 h^–1^) was found to be approximately five times that of PtB/SiO_2_ (0.05 h^–1^) catalyst, indicating remarkable resistance to deactivation achieved by the presence of B on the catalyst.


Eq. 1 $$K_{d}=[ln((1-x_{end})/x_{end})-ln((1-x_{start})/x_{start})]/t$$

PtB/SiO_2_’s selectivity to propylene, shown in Figure 5b, was found to be 87%, slightly higher than that of Pt/SiO_2_ with a propylene selectivity of 80%. Both selectivity profiles remained nearly unchanged during the reaction duration. Similar selectivity profiles indicate that the presence of B does not alter the reaction mechanism of propane dehydrogenation significantly.

Propane conversion profiles shown in Figure 5a were obtained in experiments where the catalyst amount was kept the same at 200 mg. Pt loading of PtB/SiO_2_ (0.05%) and PtB/SiO_2_ (0.11%) is, however, different and could cause the observed difference in propane conversion over the catalysts. To take the difference in Pt amount into account, the rate of propylene production was calculated at equal amount of Pt and presented in Figure 5c. The results show that the initial rate of propylene production over the catalysts is similar, indicating that the initial conversion difference shown in Figure 5a stems from the different amounts of Pt present in each reactor. In other words, the activity of fresh catalyst per amount of Pt is nearly the same. However, during the reaction, PtB exhibited a more stable profile than Pt. At the end of the reaction, the rate of propylene formation achieved over PtB was found to be 2.7 times that of Pt, indicating remarkable enhancement of catalytic performance by boron incorporation into the Pt/SiO_2_ catalyst.

Elemental analysis was performed over the samples recovered from the reactor after the catalytic activity experiment was over. Pt content of Pt and PtB/SiO_2_ was measured using ICP-OES as 0.05% and 0.10%, respectively. B content of PtB/SiO_2_ was measured as 0.64%. Elemental analysis did not reveal any meaningful differences in metal content between the fresh and used catalysts, indicating that the activity loss cannot be attributed to the loss of the metals, via leaching or diffusion into the reactor material.

The results of this study showed that PtB catalyst is more selective and stable than Pt catalyst for nonoxidative propane dehydrogenation. The enhancement in the propylene selectivity was attributed to the presence of electron-rich Pt sites that favored desorption of propylene and prevented over-dehydrogenation, which in turn, affected the stability of the catalysts [12]. The most likely cause of deactivation under the reaction conditions is the formation of coke that covers the active sites and decreases the catalytic performance. The role of Boron is to alleviate this effect and improve the catalytic performance, stability in particular. Boron modification of inorganic metal oxides was found to improve the stability of dehydrogenation of other light alkanes by mitigating coke formation in butane dehydrogenation [31,33] and preventing the sintering of active sites [31]. Byron et al. reported that the migration of carbonaceous deposits forming on Pt sites was facilitated in the presence of tetrahedrally coordinated boron sites on the surface. In other words, boron scavenges carbonaceous deposits and improves the stability of the catalysts. Dadras et al. found that the presence of boron on the surface decreases the adsorption of carbonaceous deposits on Pt sites by density functional theory calculations [31]. The same group also reported that the presence of boron decreases the sintering of Pt sites by Ostwald ripening [31].

## 4. Conclusions

Sequential deposition of Pt and B on SiO_2_ was successfully performed via ALD of organometallic precursors in a viscous flow reactor with an intermediate calcination step between the depositions. The presence of B_2_O_3_ on the surface created new anchoring sites since more Pt sites were deposited on B/SiO_2_, as compared to bare SiO_2_. Characterization results revealed that presence of B increased the electron density of Pt sites and retarded the hydrogenolysis step of Pt sites by circa 100 °C by creating stronger interactions between the Pt precursor and B-incorporated SiO_2_. Nonoxidative propane dehydrogenation catalytic activity measurements showed that B-incorporated sample performed better than Pt/SiO_2_ with higher selectivity and remarkable stability. The enhancement of catalytic activity was attributed to more facile propylene desorption from the surface and a decrease in the formation of coke precursor/carbonaceous deposits. The resistance to deactivation was improved, with an increase in the deactivation constant from 0.24 h^–1^ to 0.05 h^–1^. All the investigations presented in this study pointed out that boron could be an excellent choice for the second metal for bimetallic catalysts and may be used to enhance catalytic performance, particularly stability.

## Figures and Tables

**Table:** Elemental composition of and Pt/SiO_2_ and PtB/SiO_2_*.

	Pt (wt%)	B (wt%)
**Pt/SiO** **_2_**	0.05	-
**PtB/SiO** **_2_**	0.11	0.68

* by ICP-OES
